# RETRACTION: ERK1/2‐Dependent Inhibition of Glycolysis in Curcumin‐Induced Cytotoxicity of Prostate Carcinoma Cells

**DOI:** 10.1155/bmri/9878760

**Published:** 2025-12-19

**Authors:** BioMed Research International

RETRACTION: Y‐J. Lee, S‐H. Lee, “ERK1/2‐Dependent Inhibition of Glycolysis in Curcumin‐Induced Cytotoxicity of Prostate Carcinoma Cells,” *BioMed Research International*, 2022, 7626405, https://doi.org/10.1155/2022/7626405

The above article, published online on 24 August 2022 in Wiley Online Library (http://wileyonlinelibrary.com), has been retracted by agreement between the journal Associate Editor, Jinsong Ren, and John Wiley & Sons Ltd. The retraction has been agreed due to multiple errors in the figures, initially identified on PubPeer [1]. On further investigation, additional concerns were identified. Specifically:

• In Figure 1b, the second and fifth HPrEC bands appear highly similar
• In Figure 2c, the *β*‐actin bands for PC‐3AcT and DU145AcT bands (top panels) appear highly similar
• In Figure 4c, there is unexpected similarity between the PC‐3AcT/CCM+/PD+ panel and the PC‐3AcT/CCM‐/PD+ panel
• In Figure 4c, there is unexpected similarity between the DU145AcT/CCM+/PD+ panel and the PC‐3AcT/CCM‐/PD+ panel
• In Figure 4c, there is unexpected similarity between the DU145AcT/CCM‐/PD+ panel and the DU145AcT/CCM+/PD‐ panel

After assessment of the concerns and the author’s response, the editorial board has determined that the results and conclusions are unreliable, and the article is therefore retracted.

The authors agree with the retraction.

## References

[1] Indigofera tanganyikensis , ERK1/2-Dependent Inhibition of Glycolysis in Curcumin-Induced Cytotoxicity of Prostate Carcinoma Cells, PubPeer. (2022) https://pubpeer.com/publications/E8247DA41B70CC721AEBA8468D4466. 10.1155/2022/7626405 PMC9433281 36060138

